# Enhancing Label-Free Fluorescence Lifetime Imaging for Intraoperative Tumor Margin Delineation in Head and Neck Cancer using Data-Centric AI

**DOI:** 10.21203/rs.3.rs-6673642/v1

**Published:** 2025-05-23

**Authors:** Mohamed Abul Hassan, Lisanne Kraft, Muhammad Adeel Azam, Julien Bec, Kelsey T Hadfield, Jinyi Qi, Dorina Gui, Arnaud Bewley, Marianne Abouyared, Jonathan Sorger, Gregory Farwell, Andrew C. Birkeland, Laura Marcu

## Abstract

Precise intraoperative delineation of tumor margin is critical for maximizing resection completeness and minimizing recurrence in head and neck cancers (HNC). Label-free Fluorescence Lifetime Imaging (FLIm), which captures the fluorescence decay characteristics of endogenous molecules, offers a real-time method for differentiating malignant from healthy tissue during surgery without the need of contrast agents. Here, we present a data-centric artificial intelligence (AI) framework to enhance the robustness and accuracy of FLIm-based classification models for HNC. FLIm data were collected in vivo from 92 patients undergoing both transoral robotic surgery (TORS) and non-TORS procedures for HNC using a multispectral FLIm device with 355 nm excitation. To improve model performance, a data-centric approach leveraging confident learning was implemented to identify and exclude instances with low quality. An interpretability framework was further integrated to quantify feature contributions and elucidate FLIm-derived sources of contrast. Under a leave-one-patient-out cross-validation scheme, the model demonstrated a strong discriminative ability with an area under the receiver operating characteristic curve of 0.94 in differentiating healthy versus cancerous tissue. In tumor boundary regions, borderline predictions revealed transitional tissue properties, with strong correlations observed between model predictions and FLIm parameters in spectral channels corresponding to NADH and FAD—key metabolic cofactors indicative of cellular metabolic shifts at tumor margins. The impact of tumor anatomical site (base of tongue, palatine tonsil, oral tongue) and p16+ (HPV) status on classification performance was also assessed. These findings underscore the potential of label-free FLIm to provide accurate, real-time guidance for intraoperative margin assessment and dysplasia grading, advancing surgical precision in head and neck surgical oncology.

### I. Introduction

Accurate intraoperative delineation of tumor margins in head and neck cancers (HNC) is critical for achieving complete resection, reducing the risk of recurrence, and preserving critical structures to minimize morbidity[1,2]. Among the emerging optical imaging modalities, label-free fluorescence lifetime imaging (FLIm) has shown significant potential in identifying HNC within the oral cavity and oropharynx, establishing itself as a valuable tool for intraoperative surgical guidance[3–7]. In contrast to alternative image-guided surgery approaches currently used in HNC surgery[8], which rely on the preoperative administration of near-infrared (NIR) fluorescent molecular probes such as indocyanine green (ICG)[9], 5-aminolevulinic acid (5-ALA)[10], or IRDye800CW[11], label-free FLIm utilizes the intrinsic fluorescence decay properties of endogenous tissue components, such as Nicotinamide Adenine Dinucleotide (NADH), Flavin Adenine Dinucleotide (FAD), and collagen, FLIm, to differentiate pathological from normal tissues based on their distinct lifetime signatures. Label-free FLIm enables in-situ real-time imaging without the use of exogenous contrast agents, allowing tissue characterization based on intrinsic biochemical and functional properties.

Moreover, recent advancements in fiber optic-based, label-free FLIm systems have significantly improved surgical flexibility and precision, allowing navigation of complex anatomical regions either manually using a hand-held probe or through a transoral robotic surgical (TORS) system during tumor resection[14,15]. This adaptability is especially critical in the anatomically complex and variable landscapes of head and neck surgeries[16], offering a distinct advantage in navigating the intricate regions encountered in HNC procedures.

While label-free FLIm is adaptable to a variety of clinical scenarios and tissue autofluorescence offers rich intrinsic information, accurate interpretation in relation to pathology requires robust analytical approaches. Classification models based on FLIm-derived parameters have been developed as a tool to enhance cancer delineation, enabling real-time surgical guidance[17,18]. However, the robustness of these models is often challenged by the inherent heterogeneity of HNC, which is further compounded by biological factors such as HPV status (e.g., p16-positive HNC) and anatomical variations in tissue autofluorescence across different subsites (e.g., base of tongue, tonsil, or superior tongue)[19–21]. Additionally,

This work was supported by the National Institutes of Health (Grants: 2R01CA187427 and P41 -EB032840-01). Mohamed Abul Hassan, Lisanne Kraft, Muhammad Adeel Azim, Kelsey T Hadfield, Julien Bec, Jinyi Qi, work with the Department of Biomedical Engineering, University of California Davis, USA. Andrew C. Birkeland Arnaud Bewley, Marianne Abouyared, practice oncologic surgery within the Department of Otolaryngology – Head & Neck Surgery, University of California Davis, USA. Joathan Sorger is with Intuitive Surgical, Sunnyvale, USA. D. Gregory Farwell is with Department of Otorhinolaryngology – Head and Neck Surgery, University of Pennsylvania, Davis, USA. Laura Marcu is with the Department of Neurological Surgery and the Department of Biomedical Engineering, University of California Davis, USA. Corresponding Author Laura Marcu (e-mail: lmarcu@ucdavis.edu).

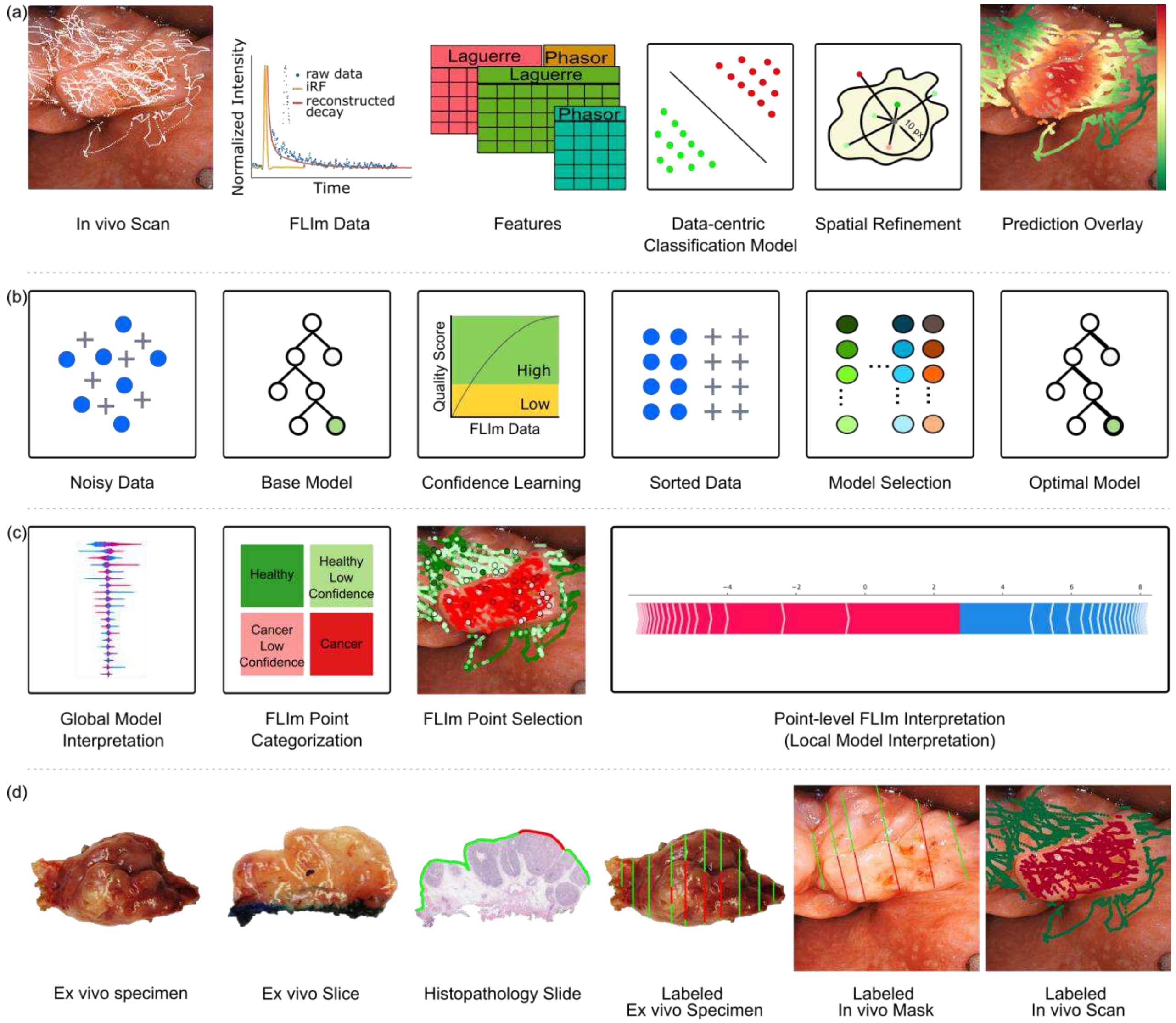

Fig. 1 Overview of the methodology for label-free FLIm-based intraoperative surgical guidance, data collection, histopathology registration, and data analysis. **Panel (a):** An overview of the FLIm-based, data-centric classification model developed for in vivo prediction of head and neck cancer (HNC). **Panel (b):** The development of the data-centric model using confidence learning to sort high quality data from noisy data for model training. **Panel (c):** Investigation of both global and local FLIm feature contributions through SHAP (SHapley Additive exPlanations) analysis and data-centric instance categorization (The circled points are the instances that is selected through the data centric approach and lighter gradient indicates low confidence data). **Panel (d):** The labeling workflow for model training and testing, with labels derived directly from histopathology and reviewed by the clinical pathologist (DG). Each annotated H&E section was co-registered with the corresponding ex vivo and in vivo FLIm scan. Red annotations represent cancerous tissue, while green annotations represent healthy tissue.

inconsistencies in data acquisition, arising from variations in scanning techniques and discrepancies in the co-registration between histopathology and in vivo measurements, pose further challenges to achieving reliable classification outcomes[22]. To effectively support intraoperative decision-making, these models must address such sources of variability and demonstrate a high degree of robustness.

This study presents a data-centric (DC) framework aimed at improving the reliability of label-free FLIm-based classification models for HNC. The inherent variability in FLIm data from HNC tissues complicates the model's ability to establish reliable decision boundaries, increasing the risk of overfitting to noise or spurious patterns. The DC approach described here systematically identifies and removes data points with low confidence or potential labeling issues that arise from these variabilities. By focusing on high-quality, consistent data, the framework minimizes the impact of confounding factors, thereby improving the overall stability of the model. This enhanced stability enables the model to maintain consistent performance under the complex and

Table. I Global analysis of classification efficacy for discriminating healthy vs. cancerous tissue at point-level and region-level using a data-centric classification model on in vivo FLIm scans. The analysis included n = 677,821 FLIm points from 92 patients. To address dataset imbalance across anatomical regions, optimal thresholds were computed as follows: Base of Tongue (0.24), Palatine Tonsil (0.24), and Oral Tongue (0.44)

| Anatomy | Point-level | | | Region-level | | |
|---|---|---|---|---|---|---|
| | AUC | Sensitivity | Specificity | AUC | Sensitivity | Specificity |
| Base of tongue (N = 24 \| n = 180,334) | **0.80** | 0.73 | 0.73 | **0.84** | 0.76 | 0.80 |
| Palatine tonsil (N = 27 \| n = 247,779) | **0.89** | 0.82 | 0.81 | **0.96** | 0.92 | 0.88 |
| Oral tongue (N = 41 \| n = 293,978) | **0.90** | 0.84 | 0.81 | **0.93** | 0.90 | 0.81 |
| Overall (N = 92 \| n = 667,821) | **0.89** | 0.83 | 0.82 | **0.94** | 0.91 | 0.85 |

variable conditions encountered during intraoperative imaging.

As summarized in Fig. 1, FLIm data is acquiring in vivo from multiple anatomical sites (i.e. base of the tongue, palatine tonsil and oral tongue) prior to tumor resection. These point-by-point FLIm measurements are then linked to histopathology-derived ground-truth labels through a co-registration process that spatially matches each FLIm signal to the corresponding region on the excised tissue specimen. Following standard grossing and formalin-fixation, the resected specimen is sectioned, H&E-stained, and digitally annotated by the clinical pathologist (DG).

A confidence-learning algorithm[23] was applied to systematically assess data quality and exclude ambiguous or low-confidence FLIm points. As illustrated in Fig. 1 (panel b), this approach identifies problematic data that can arise from shifts in tissue metabolic state (manifesting as changes in autofluorescence signals near tumor margins) or acquisition challenges (e.g., suboptimal probe orientation) and removes these points from the training set. This process generates a high-fidelity dataset composed exclusively of high-confidence FLIm points that more accurately reflect overall tissue properties. Our results show that this targeted data selection enhances the model's ability to distinguish malignant (squamous cell carcinoma) from healthy tissues under heterogeneous scanning conditions and across multiple anatomical sites.

### II. Results

The classification model based on the DC approach was evaluated using FLIm data collected from both healthy and cancerous tissues. The dataset, consisting of 677,821 FLIm points from 92 patients across three anatomical regions, the base of tongue, palatine tonsil, and oral tongue, was curated to maximize data quality. Model evaluation was performed using a leave-one-patient-out cross-validation (LOPO-CV) scheme, ensuring that each test set remained independent and unaltered. This approach provides an unbiased assessment while closely reflecting real-world clinical conditions.

The classification performance evaluated at both point and region levels capturing prediction at intendent point-level and aggregated as well as refined predictions at local tissue level are depicted in Table. I. At the point level, the model demonstrated strong discrimination power between healthy and cancerous tissues, achieving an overall area under the receiver operating characteristic curve (AUC) of 0.89. Palatine tonsil and oral tongue regions reported higher AUC values (0.89 and 0.90, respectively), with balanced sensitivity (0.82–0.84) and specificity (0.81–0.82). While the base of tongue reporting a slightly lower point-level AUC of 0.8, the performance remained competitive.

At the region-level, the model's efficacy improved substantially. Across all anatomies, the region-level AUC consistently surpassed the point-level performance, reaching an overall AUC of 0.94. This improvement was most pronounced for the palatine tonsil, where the AUC increased from 0.89 at the point-level to 0.96, alongside marked improvements in sensitivity and specificity. The base of the tongue regions also benefited from region-level refinement with the AUC increased from 0.78 to 0.84. These findings suggest that incorporating spatial information and aggregating predictions over tissue areas can mitigate measurement variability and result in a more robust classification outcome.

#### A. Models Comparison and Benchmarking

To evaluate classification performance across different training strategies, three models were compared: the data-centric (proposed approach), the agnostic model (i.e., trained on the full data without filtering) and the anatomy-specific model[21] (i.e., trained with region-specific information). The data-centric model achieved the highest overall classification performance, with an AUC of 0.94, compared to 0.86 and 0.85 for the anatomy-specific and agnostic models, respectively. Analysis of patient-level AUC distributions showed that 81.97% of patients achieved an AUC greater than 0.8, with most cases approaching near-perfect classification (close to 1.0) (Figure 2, AUC distribution histogram).

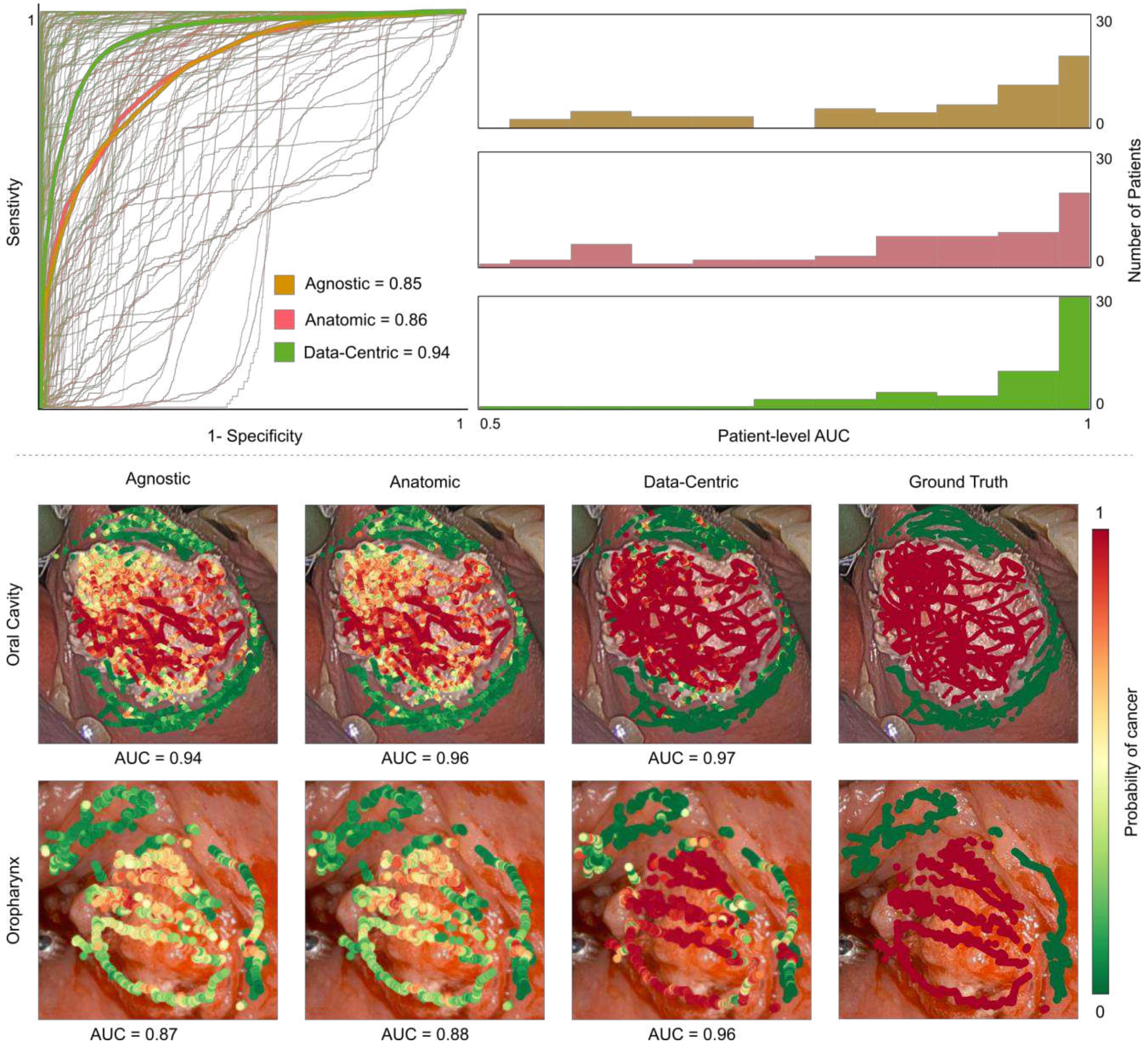

Fig. 2 Illustration of the model's performance and the influence of data-centric classification. **Upper Panel**: Overall analysis using ROC curves along with the patient-level AUC distributed as histogram for three approaches—agnostic, anatomic, and data-centric models. The thicker line denotes the global ROC curve, while the thinner lines represent patient-level ROC curves for each model subset demonstrating the breadth of performance across models. **Lower Panel**: Representative intraoperative images (top: oral cavity; bottom: oropharynx) with color-coded overlays depicting localized cancer probability predictions. Colors range from green (healthy) to red (cancer), indicating the model's estimated likelihood of malignancy at each sampled location.

To visualize point-level prediction efficacy, representative intraoperative images from both oral cavity and oropharynx procedures were overlaid with cancer probability predictions for each model (Figure 2 right panel). At the point level, a key observation is the bi-modal distribution in the data-centric model's predictions, where cancerous and healthy regions are classified with high confidence. In contrast, the agnostic and anatomic models predominantly yield borderline cancer predictions, demonstrating lower confidence in distinguishing between healthy and cancerous regions. This improvement in classification confidence further underscores the advantage of a data-centric training approach, enabling more precise and clinically interpretable intraoperative predictions.

### B. FLIm Feature Contribution

A grid search-based approach was employed for model and feature selection, revealing that the highest classification performance was achieved using a combination of average lifetime, intensity ratio, and Laguerre coefficients values from the three spectral channels (see Supplementary Fig. S1). This

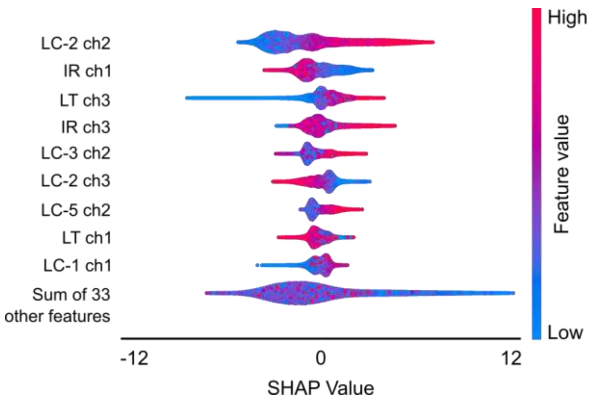

Fig. 3 SHAP summary plot illustrating the overall feature contributions of the data-centric model across the entire dataset. Each violin represents the distribution of SHAP values for one feature, ranked by importance from top (most significant) to bottom (least significant). A positive SHAP value indicates a feature's contribution toward a cancer classification, whereas a negative value indicates a contribution toward a healthy classification. The color scale (blue to pink) denotes low to high original feature values, respectively. *LT – Average lifetime | IR – intensity ratio | LC – Laguerre coefficient*

trend was consistent across both the agnostic and anatomic-specific model training strategies. These spectral channels (i.e., 390/40 nm (Channel 1), 470/28 nm (Channel 2), and 542/50 nm (Channel 3) primarily capture autofluorescence from collagen, NADH, and FAD, respectively, each depicting distinct biochemical processes within the tissue.

A global analysis of the data-centric model was performed by aggregating the absolute Shapley values across all FLIm points[24]. The SHAP summary plot (Fig. 3) ranks feature by their contribution to the model's predictions, with the most influential listed at the top. Among all FLIm-derived parameters, the second-order Laguerre coefficient from Channel 2 (LC-2), corresponding to the NADH-dominant spectral region, emerged as the most significant. LC-2 reflects the weighting of the second Laguerre basis function in the fluorescence decay curve, emphasizing early-time decay components. Since free NADH exhibits a rapid decay (<1 ns) compared to its protein-bound form (>1.0 ns)[25], an elevated free-to-bound NADH ratio—common in malignant cells—results in a faster decay and higher LC-2 values, thereby biasing model outputs toward a "cancer" classification. In contrast, lower LC-2 values, indicative of slower decay dynamics, are characteristic of healthy tissue. These findings are consistent with prior reports[26].

In contrast, the intensity ratio from Channel 1 (associated with collagen fluorescence) showed the opposite trend. Lower ratios were linked to cancer, aligning with the known degradation or remodeling of the extracellular matrix in tumorigenesis, while higher ratios signified healthy tissue. Additionally, in Channel 3 (FAD-dominant), shorter average lifetime values driven by a prevalence of protein-bound FAD characterizing healthy tissue, whereas longer lifetimes reflecting increased contributions of free FAD in malignant regions[27]. This shift in the bound-to-free FAD ratio underlies the elevated Channel 3 lifetime in head and neck cancers, providing robust, label-free contrast for model discrimination of healthy tissue from cancer.

#### C. Performance Variation Across Models

To further examine classification variability across models, an extended analysis was performed on a patient whose cancer-probability predictions differed significantly among the evaluated FLIm models. Local interpretation was subsequently applied to a subset of indexed points selected based on a label quality score derived from the data-centric approach, using a quartile-based subsampling strategy to identify potential label inconsistencies. Fig. 4 presents spatial classification maps highlighting the selected FLIm points used for local interpretation. The lower panel illustrates feature contributions for these points using the local interpretability approach.

Points A and B originate from cancerous regions, whereas C is from a healthy region. The data-centric model assigned higher prediction scores to the cancerous points compared to the agnostic and anatomic models, which predicted either borderline or low probability scores. This aligns with FLIm feature contributions. Although the FLIm feature values remain the same across these models, the weighting of their importance varies significantly. The data-centric model assigns greater emphasis to the most discriminative FLIm features, thereby enhancing overall classification performance.

Another key observation is the role of average lifetime values from channel 3, typically associated with FAD fluorescence, in cancer classification. Although global interpretability analysis across the entire cohort ranked this feature as the third most important, associated with healthy classifications with shorter lifetimes. This patient shown that longer average lifetime values in channel 3 significantly contributed to the identification of cancerous points. This highlights the context-dependent nature of feature contributions in the model's predictions.

For point C, located in a healthy region, all models predicted a lower probability as the classifier assigns lower probabilities with a healthy classification. Notably, the anatomic and data-centric models generated the lowest probability scores, further supporting the overall healthy classification across classification maps. This finding underscores the importance of training strategies that account for the inherent heterogeneity of FLIm data in HNC.

This case study underscores the variability in classification confidence across models, especially in regions with high heterogeneity at the margins of cancerous tissues. The

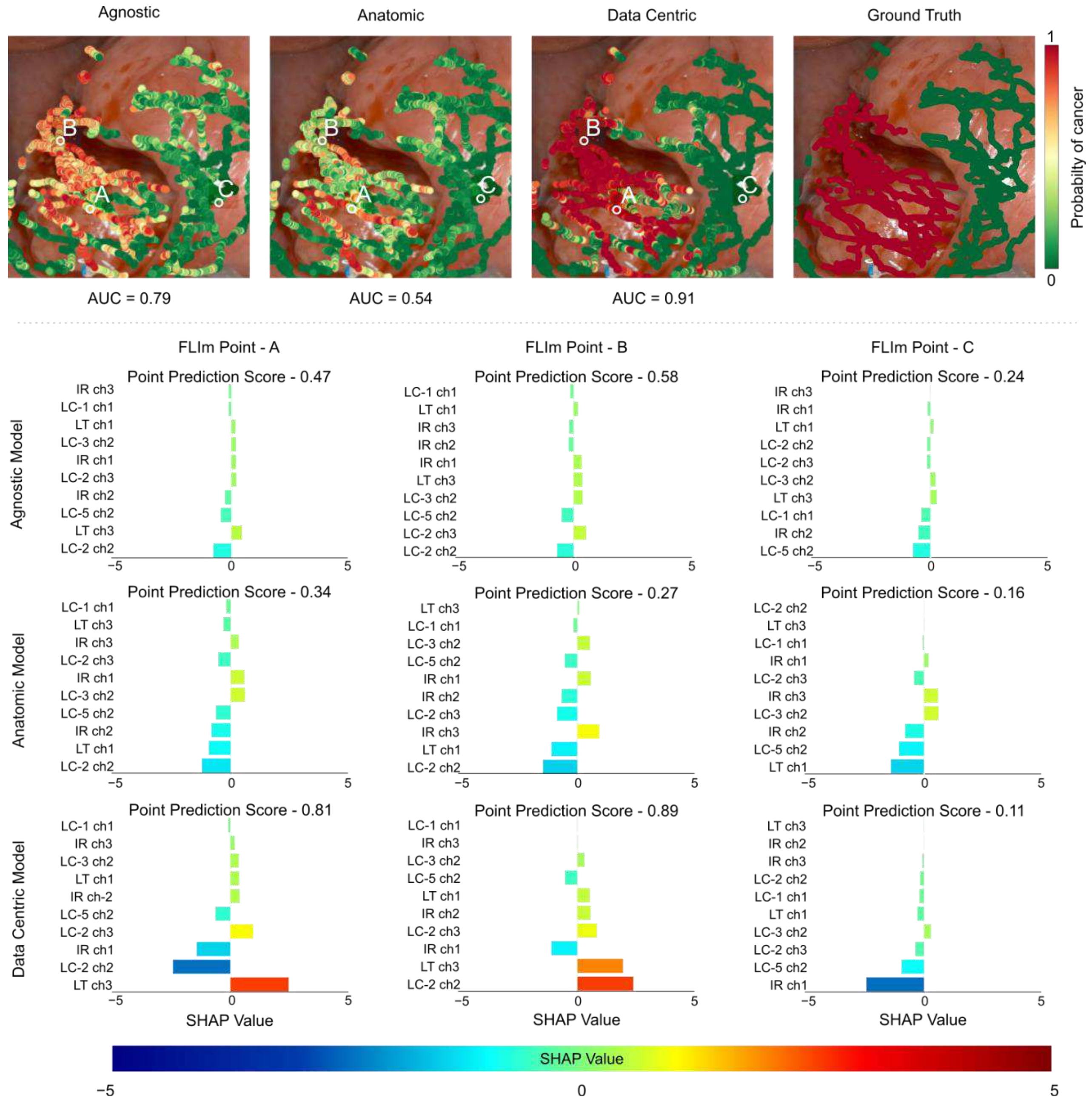

Fig. 4 Illustration of the model's variability for a specific patient. **Upper panel**: Overall classification performance (AUC) across different model configurations, overlaid on the patient's lesion. Points A and B mark cancerous regions, whereas point C indicates healthy tissue. **Lower panel**: Point-level performance for the three representative points: A, B, and C using our local interpretability (Shapley value) approach (see Methods II.G). The bar charts highlight how individual features influence the model's prediction at each labeled point, with colors ranging from blue (negative contribution toward a healthy prediction) to red (positive contribution toward a cancer prediction). Features in each bar chart are organized by their overall feature significance, with the most significant features placed at the bottom. *LT – Average lifetime | IR – intensity ratio | LC – Laguerre coefficient*

observed discrepancies, driven by inconsistent feature weighting and context-dependent variations in FLIm data, indicate that variability exists not only across the broader patient cohort but also among individual point measurements within the same patient. These findings underscore the need for a robust classification model that can reliably capture FLIm contrast, thereby enabling more consistent and accurate classification outcomes.

### D. Influence of Biological Features on Model Performance

To evaluate the impact of biological factors on classification efficacy, model performance was analyzed across different anatomical regions and patient-specific attributes. Fig. 5

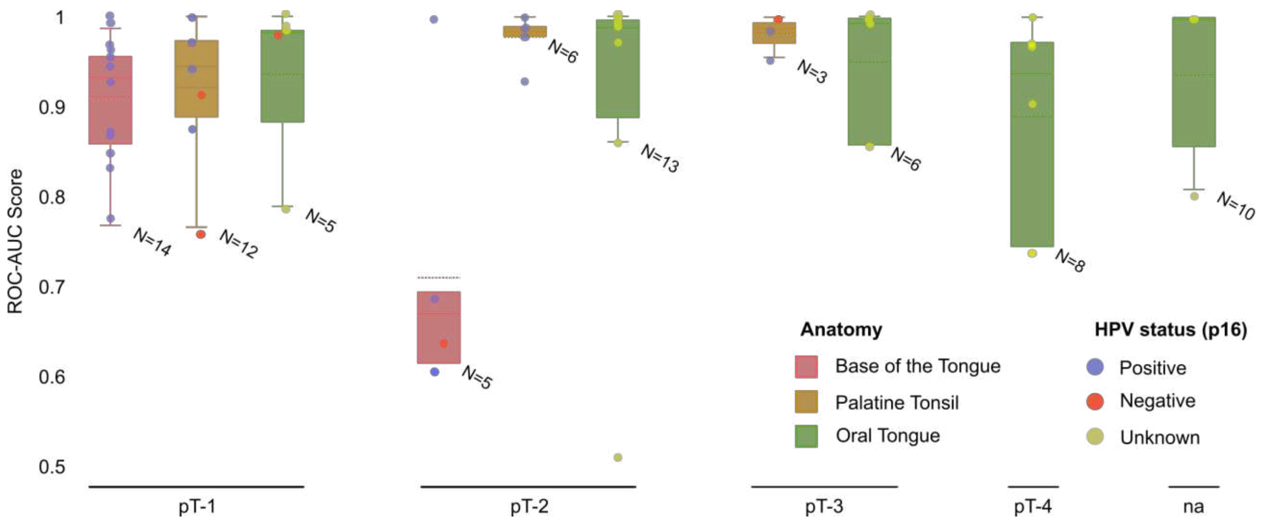

Fig. 5 Influence of tumor stage, anatomical location, and HPV (p16) status on model efficacy. The boxplots illustrate model performance across different pT classifications (pT0–pT4), anatomical sites within the head and neck region, and p16 (HPV) status. The pT classification represents tumor size and extent: T1 means < 2 cm tumor, T2 2-4 cm, T3 > 4 cm, and T4 extensive into adjacent bone or other critical tissues.

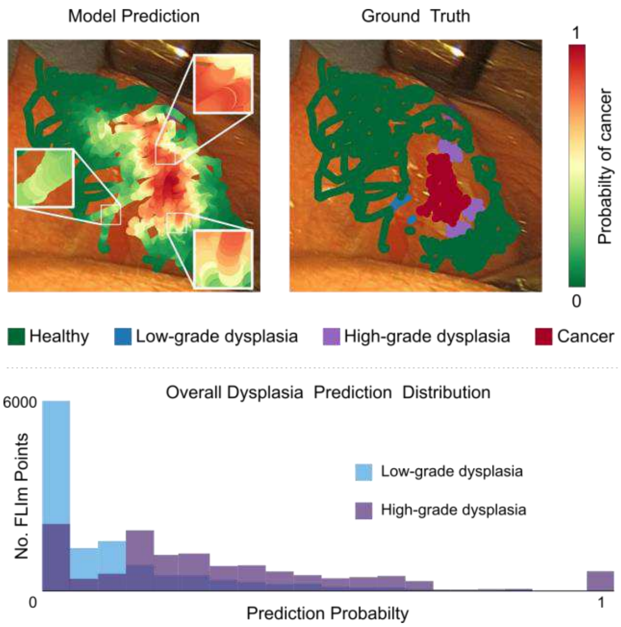

Fig. 6 Model efficacy on dysplasia classification and prediction distribution. **Upper panel**: The left image illustrates the model's classification output, while the right image represents the ground truth, with regions labeled as healthy (green), low-grade dysplasia (blue), high-grade dysplasia (purple), and cancer (red). Regions between healthy and cancer that are not labeled are excluded by the boundary exclusion filtering parameter to remove ambiguous annotations. Insets highlight prediction variations among low-grade dysplasia and high-grade dysplasia. **Lower panel**: The histogram presents the overall prediction distribution, illustrating the frequency of low-grade dysplasia (blue) and high-grade dysplasia (purple) predictions across the dataset (N = 21, n = 25,110). The model effectively captures progressive transitions from healthy tissue to dysplastic and cancerous regions, aligning with evolving tissue changes associated with dysplasia.

presents a comparative evaluation, highlighting the variation in classification performance across anatomical sites, HPV status, and pathological tumor staging.

Across anatomical regions, the base of the tongue showed the lowest classification performance, whereas the palatine tonsil achieved the highest (see Table I). HPV status was also examined, with most oropharyngeal cancer patients being HPV-positive. However, model performance did not significantly differ between HPV-positive and HPV-negative cases (Fig. 5).

The pathological primary tumor stage (pT) classification showed associations to model efficacy. Patients with pT-2 tumors at the base of the tongue were among the lowest-performing cases (Fig. 5). A similar trend was observed in oral tongue cases, where advanced tumor stage correlated with greater variability in model performance. This may reflect alterations in fluorescence properties due to changes in the tumor microenvironment at advanced stages.

#### E. Evaluating Model Performance on Dysplastic Tissue

In addition to healthy tissue and cancer regions, the pathologist also annotated areas of both low- and high-grade dysplasia (low grade dysplasia: low rates of malignancy | high grade dysplasia: high rates of malignancy). For p16+ oropharyngeal tumors, dysplasia was not annotated in accordance with American Joint Committee on Cancer (AJCC) guidelines, as Waldeyer's ring lacks a continuous basement membrane[28]. However, for p16- oropharyngeal tumors and tumors of the oral cavity, dysplastic regions were annotated by the pathologist.

To evaluate the model's ability to distinguish between low-grade and high-grade dysplasia, the data-centric model,

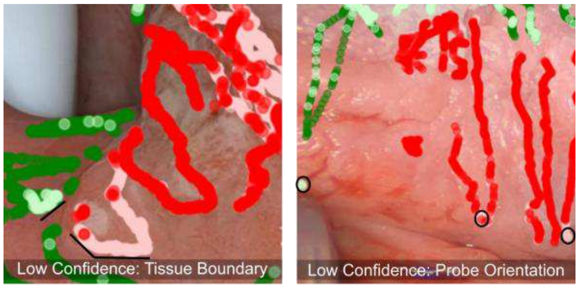

Fig. 7 Illustration of potential sources of low confidence data. **Left**: examples at the tissue boundary, annotated by the black line. **Right**: example of low confidence data due to sub optimal prob orientation, annotated by the black circles.

trained exclusively on FLIm data from healthy and cancerous tissue, was tested on FLIm measurements obtained from oral tongue tissue of 21 patients. When low-grade dysplasia was grouped with healthy tissue and high-grade dysplasia with cancer, the model achieved an AUC of 0.91. Predictions for low-grade dysplasia were primarily clustered near 0, whereas predictions for high-grade dysplasia showed a broader prediction distribution, with values clustering around 0.4 and 1.0 (Fig. 6).

### III. Discussion

This study demonstrates that integrating a data-centric AI framework with FLIm enhances the stability and generalizability of cancer-versus-healthy tissue classification across HNC sub-sites. By systematically identifying and excluding noisy or low-confidence measurements, the model leverages high-fidelity, robust data. Additionally, confidence-based selection of FLIM points improves the model interpretability, highlighting key spectral features—particularly NADH- and FAD-related signals—that most effectively distinguish cancerous from healthy tissue. These advancements underscore the potential for more precise intraoperative guidance, enhancing surgical margin assessment and ultimately improving patient outcomes in head and neck surgical oncology.

#### A. Data Centric Artificial Intelligence

The data-centric approach based on confidence learning categorizes FLIm data into high-confidence and low-confidence subsets. Low-confidence assignments are not limited to errors in pathology co-registration; they may also arise from variations in data acquisition protocols, instrument noise, and inherent tissue heterogeneity.

Across the dataset, 21.52% of FLIm points were identified as low confidence, with 19% of healthy tissue points and 27% of cancerous tissue points flagged. Low confidence data varied by patient and did not follow a consistent overall trend. However, low confidence data were more frequently observed at the periphery of the scan regions and at the interface between cancerous and healthy tissue, where FLIm signatures differed from those in the central scan regions (Fig. 7). This pattern likely reflects FLIm's sensitivity to biochemical and metabolic changes that occur at tumor boundaries.

Further analysis of surgical videos (Fig. 7) revealed that suboptimal probe orientation during data acquisition also contributed to low-confidence points, presumably due to inconsistent light delivery and collection geometry. These observations underscore the value of standardized scanning protocols and carefully curated data, both of which are essential for minimizing classification uncertainty and enhancing the reliability of AI-driven analysis.

From a modeling standpoint, label noise distorts pseudo-residuals (i.e., approximations of the first- and second-order derivatives of the loss function) during the training of decision tree-based classifier. Such distortions can result in suboptimal splits, weakening the model's ability to define meaningful decision boundaries and increasing susceptibility to overfitting to erroneous patterns. By filtering out instances with low confidence, the model achieves more stable gradient estimates, leading to more reliable split thresholds and improved leaf assignments. While this process reduces the overall training set size, it enhances model generalization by prioritizing high-fidelity data over noisy or misleading samples.

#### B. Factoring in Human, Biological, and Instrumental Variability

This study included procedures performed by four different surgeons, yet no significant association was observed between model performance and the operating surgeon (see Supplementary: Fig. S2 & S3) suggesting minimal impact of inter-surgeon variability on classification performance. In addition, scan speed during surgery, which ranged from 20 to 100 pixels per second, showed no association with fluctuations in model predictions. The high sampling rate of the FLIm system (460 Hz) likely mitigates the effects of scanning velocity variations, ensuring consistent predictive performance.

To minimize instrument-related noise in the FLIm system, potential fiber autofluorescence detected in channel 1 was removed by background subtraction and applying a gain threshold of $\leq 350$.[29] Additionally, spatial refinement techniques integrating inverse distance weighting and signal-to-noise ratio (SNR)-based adjustments were implemented to compensate for scanning variability and mitigate the impact of system noise on point-level prediction.

Notably, reduced model performance was observed in base-of-tongue cases at pathological tumor stage pT-2, while palatine tonsil cases at the same stage demonstrated higher performance. Although these anatomical sites share similar

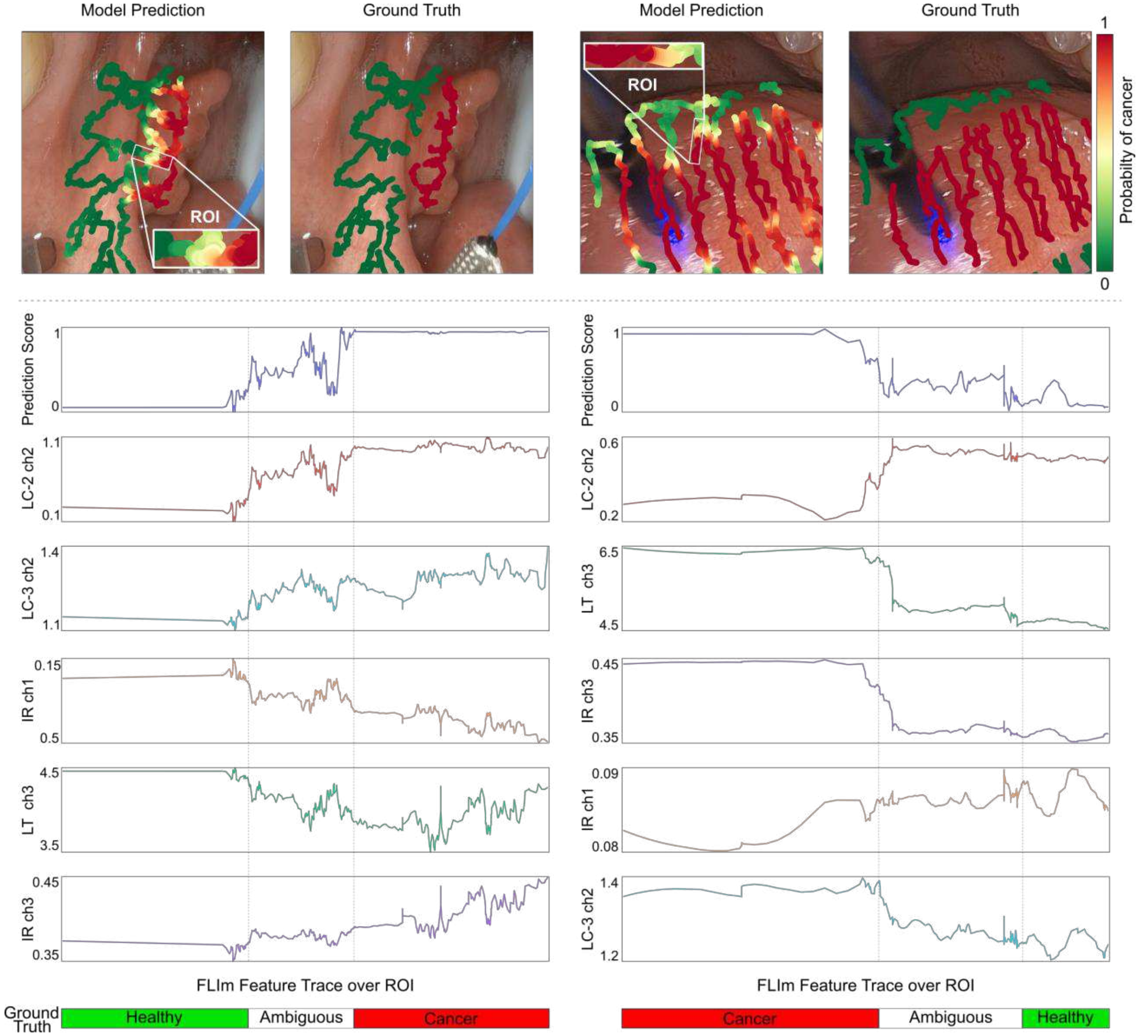

Fig. 8 Evaluation of model predictions at tumor boundaries. **Upper panel**: Representative cases from oropharynx (left) and oral cavity (right) procedures, displaying model predictions overlaid on intraoperative images. Ground Truth: Green and red annotations denote healthy and cancerous regions, respectively; the unannotated region between these areas is intentionally excluded from analysis due to ambiguous histopathology co-registration. The insets highlight borderline predictions within these ambiguous boundary regions. **Lower panel**: FLIm feature traces across the region of interest (ROI) illustrate the relationship between fluorescence-derived parameters and model predictions. Features are arranged by patient-level model interpretability ranking, with the most significant feature at the top. The traces are generated by transforming the spatial representation of FLIm points within a 5-pixel window (ROI) into a one-dimensional trace, which is subsequently smoothed using a 5-sample window Savitzky–Golay filter. The Y-axis is presented in arbitrary units (a.u.) for all FLIm features, except for average lifetime, which is measured in nanoseconds (ns). Refer to supplementary Fig. S4 for an illustration of the spatial distribution of FLIm points within the ROI. The observed correlation between FLIm feature traces and model predictions underscores FLIm's sensitivity to metabolic changes at tumor margins, reinforcing its potential for intraoperative margin delineation.

biochemical and morphological features, tumor stage alone may not explain the observed discrepancy. The lower model performance in the base-of-tongue region may also be attributed to challenges in scanning and data acquisition, as indicated by the reduced number of FLIm points collected at this site (see Table I & Table II). This difficulty is further attributed by the anatomical location of the base of tongue, which necessitates a more tangential and less perpendicular scan compared to other oral cavity and tonsillar sites. Such an approach likely affects the mechanics of data acquisition and may contribute to the difficulty in obtaining high-quality FLIm data from this region.

#### C. Can FLIm Identify the 'Margin'?

In this study, we excluded a 15-pixel (approximately 0.55 mm) boundary between adjacent cancerous and healthy tissue regions to mitigate potential orientation/co-registration protocol challenges in the label mask definition, acknowledging the difficulty of pinpointing the exact tumor boundary. To assess the behavior of the model at this boundary, we specifically analyzed these excluded "ambiguous" regions.

As illustrated by the two representative cases (oropharynx and oral cavity) in Fig. 8, the DC model generated borderline predictions within these boundary areas, whereas its predictions were highly confident in distinctly healthy or cancerous regions. Further examination of the underlying FLIm parameter traces showed a strong correlation between Laguerre coefficient 2 (channel 2) and the model prediction in the TORS case, and a similar correlation between average lifetime (channel 3) to the model prediction in the non-TORS case. These findings are complementary as NADH and FAD are key metabolic cofactors, their fluorescence profiles, particularly fluorescence decay and lifetime are highly sensitive to cellular metabolic shifts commonly observed at tumor margins.

These findings underscore FLIm's sensitivity to metabolic changes at the tumor interface and highlight its potential utility for intraoperative margin delineation. By capturing these subtle autofluorescence changes at tissue boundaries, FLIm provides an additional layer of real-time insight that may ultimately enhance surgical decision-making and margin assessment[30].

#### D. Study Limitations and Future Directions

While the current analysis was limited by patient numbers within specific groups, the findings provide a strong foundation for future studies. As larger datasets become available, it will be possible to more thoroughly evaluate the influence of surgeon variability, biological factors, and data acquisition. Moreover, the promising predictive performance and FLIm contrast observed at tumor boundaries highlight the potential of this approach, warranting further exploration across diverse tumor pathologies and invasiveness levels.

### IV. Materials and methods

#### A. FLIm Instrumentation and Data Acquisition

This study utilized a multispectral fluorescence lifetime imaging (FLIm) device to acquire data[29]. The device employed a 355 nm UV laser for fluorescence excitation, operating at a 480 Hz repetition rate. Excitation light was delivered to the tissue via a 365 µm multimode optical fiber (0.22 NA), which also relays the collected fluorescence signal to a set of dichroic mirrors and bandpass filters to spectrally resolve the auto-fluorescence into 3 different spectral bands: 390/40 nm, 470/28, and 542/50 nm, tailored to the peak emissions of collagen, NADH and FAD, respectively. Autofluorescence is detected by three variable gain UV-enhanced silicon avalanche photodiode (Si APD) modules with integrated trans-impedance amplifiers, which capture the fluorescence signal across each spectral band. The autofluorescence waveform measurements for each channel were averaged four times, yielding 115 measurements per second at a 460 Hz excitation rate[31].

FLIm data were acquired during two distinct surgical procedures: 1) Transoral robotic surgical procedures (TORS) using the da Vinci SP for oropharyngeal cancer, and 2) non-TORS hand-assisted procedure for oral cavity cancer. The average duration of each FLIm scan was approximately 45 seconds, resulting in an average of 5,400 point measurements per surgical field. All scans were performed prior to initiation of tissue excision. In typical cases, minimal or no blood was present in the operative field. For a small subset of patients experiencing bleeding due to tongue retractors or nasal intubation, the region of interest for FLIm scanning was rinsed with isotonic saline and aspirated prior to optical scan.

The method for acquiring and visualizing the FLIm data was tailored to distinct surgical scenarios. In non-TORS procedures, a hand-held fiber optic probe with ball lens focusing optics, coupled with an endoscopic camera (Stryker), was used. In contrast, for TORS procedures, the fiber optic probe was actuated by robotic instruments, with the surgical field visualized through the integrated da Vinci camera.

The FLIm device includes a 440 nm continuous wave laser that serves as an aiming beam; this aiming beam enables real-time visualization of the locations where fluorescence (point measurements) is collected. Segmentation of the 'aiming beam' allows for FLIm data points to be localized as pixel coordinates within a surgical white light image (see Fig. 1). Localization of these coordinates in combination with tissue label masks obtained from pathological analysis[14,32]. (see Fig. 1) enables labeling of FLIm optical data.

#### B. Patient Cohort and FLIm data labeling

The research was performed under the approval of the UC Davis Institutional Review Board (IRB) and with the patient's informed consent. All patients were anesthetized, intubated, and prepared for surgery as part of the standard of care. N

Table. II Anatomy and Tissue label breakdown for the 92 patients

| Anatomy N(n) | Tissue Label | No. Patients (N) | No. FLIm points (n) |
|---|---|---|---|
| Base of tongue 24(180,334) | Healthy | 24 | 141,134 |
| | Lymphoid | 8 | 8,834 |
| | Cancer | 20 | 30,366 |
| Palatine tonsil 27(247,779) | Healthy | 27 | 195,916 |
| | Lymphoid | 8 | 10,326 |
| | Cancer | 17 | 41,537 |
| Oral tongue 41(293,978) | Healthy | 41 | 134,353 |
| | LGD | 9 | 12,738 |
| | HGD | 12 | 12,372 |
| | Cancer | 34 | 134,515 |

Table. III Description of the feature selection used for model grid-search

| Grouping name | FLIm features |
| --- | --- |
| LT | Average Lifetime |
| IR | Intensity Ratio |
| LC | Laguerre Coefficients |
| IR+LC | Intensity Ratio, and Laguerre Coefficients |
| LT+IR+LC | Lifetime, Intensity Ratio, and Laguerre Coefficients |
| PH | Phasor |
| LC+PH | Laguerre Coefficients, Phasor |
| All | Lifetime, Intensity Ratio, and Laguerre Coefficients, Phasor |

=100 patients enrolled, and eight patients excluded from analysis due to absence of in vivo scan, failed registration with tissue histopathology or instrumentation issues (e.g., malfunctioning software or damaged fiber optic probe) during procedure. N=92 patients are represented in this study, comprising HNC in the oral tongue (N=41), palatine tonsil (N=27) and the base of the tongue (N=24).

For each patient, the operating surgeon conducted an en bloc surgical tumor resection procedure, and the resulting excised specimen was sent to a surgical pathology room for grossing. The tissue specimen was serially sectioned to generate tissue slices, which were then formalin-fixed, paraffin-embedded, sectioned, and stained to create Hematoxylin & Eosin (H&E) slides for pathologist interpretation (see Fig. 1). To correlate FLIm measurements with pathology labels (e.g., benign vs. cancerous tissue), the pathologist digitally annotated each H&E section. These annotations were then mapped onto the ex vivo specimen at the corresponding cut lines and subsequently co-registered with the in vivo surgical field. This co-registration process enabled direct validation of FLIm data with the pathological status of the tissue under investigation (see Table II).

### C. FLIm preprocessing and Feature Extraction

The spectral channels 1-3 were used for analysis in this study. A gain threshold of $\leq 350$ was applied as a filtering criterion to channel 1 to select FLIm points with good signal quality (refer to supplementary Fig. S5 for analysis on gain threshold selection). The raw FLIm waveform was pre-processed for background subtraction. Two non-parametric techniques namely, Laguerre-based deconvolution[33] and lifetime phasors[34], were utilized to derive the fluorescence decay characteristics for each spectral channel. These methods generated an array of features, such as average lifetime (LT), intensity ratio (IR), 12 Laguerre coefficients (LC), and lifetime phasor (PH).

### D. Data Centric Learning

We adopt a DC framework based on the principles of confident learning to identify and remove instances with high uncertainty during the classification model development[23]. This approach focuses on improving the quality of the training data, thus enhancing model performance and robustness. The model is evaluated via a LOPO-CV scheme, ensuring that model evaluation on held-out patient's data, reflective of realistic clinical use cases.

Consider a dataset $D = \{(x_i, y_i)\}$ where each instance $x_i$ corresponds to a FLIm point from a particular patient and $y_i \in \{0,1\}$ is the binary label, potentially containing noise. To identify the potential noisy data, we use a base model: Decision Tree classification model with 100 estimators which we have reported previously [21,35] and train it on the dataset with LOPO-CV scheme. A model prediction probability $p_i$ is generated from the held-out patient's data for each FLIm point:

$$p_i = f(\hat{Y} = 1 \,|X = x)$$

Here, $\hat{Y}$ is the model's predicted label. These predicted probabilities reflect the model's assignment about how well each instance fits into the given class.

A confident joint $J_{j,k}$ is constructed using $p_i$ to capture the joint distribution of noisy (observed) labels and predicted labels for instances that the model is sufficiently certain about. Using the joint distribution, the label noise rates are estimated as:

$$\hat{\eta}_{j\to k} = \frac{J_{j,k}}{\sum_{k'} J_{j,k'}} \text{ for } j,k \in \{0,1\}$$

For $j,k \in \{0,1\}$, This approximates the conditional probability that a true class $j$ example is erroneously labeled as class $k$. These noise rates $\hat{\eta}_{j\to k}$ provide a statistical summary of labeling errors across classes, informing how prevalent and asymmetric the data noise is in the dataset.

After estimating the noise rates, we utilize instance-level measures of model-label agreement to guide the removal of potentially unreliable examples. For each instance $(x, y) \in D$, we compute a confidence score:

$$c_i = p_i \cdot \mathrm{I}(y_i = 1) + (1 - p_i) \cdot \mathrm{I}(y_i = 0)$$

where $\mathrm{I}(\cdot)$ is the indicator function and the confidence score $c_i$ reflects how well the model's predicted class probabilities support the observed label. Based on the estimated number of mislabeled instances inferred from the global noise rates, we select a threshold $\kappa = 0.4$ and retain only those instances whose confidence scores exceed $\kappa$ (refer to supplementary: Fig. S6). Formally, we construct the high-quality training subset:

$$D_{train}^{clean} = \{(x_i, y_i) \in D_{train} : c_i \geq \kappa\}$$

This data-centric pruning step aims to produce a training dataset more representative of the overall distribution with high-fidelity. Through a reduced influence of unreliable examples, the training process can focus on learning valid patterns, improving model robustness and interpretability.

We then retrain the model on $D_{train}^{clean}$ :

$$\theta_{clean} = \arg\min_{\theta} \sum_{(x_i, y_i) \in D_{train}^{clean}} \zeta\big(y_i, f(x_i; \theta)\big)$$

where $\zeta$ is the loss function, $f$ is defined as the model's predictive function, mapping input features to its output predictions and $\theta_{clean}$ is the optimal set of parameters found by minimizing the loss function over the high confidence training set.

Importantly, we do not remove or alter any instances from the test set $D_{test}$. This preserves the full complexity and potential label noise in the evaluation data, ensuring an unbiased and realistic assessment of the model's performance.

#### E. Model and Feature Selection

The selection process involved systematically evaluating a range of models and feature combinations (see Table. III) to determine the optimal configuration for classification. The models assessed included Gaussian Naive Bayes (GaussianNB), Linear Discriminant Analysis (LDA), Quadratic Discriminant Analysis (QDA), Multi-Layer Perceptron (MLP), and XGBoost Decision Tree (DT). While GaussianNB, LDA, and QDA models do not require hyperparameter optimization, Bayesian optimization was applied to fine-tune MLP and DT hyperparameters based on a patient-level grouped 3-fold cross-validation strategy (see Table SI for hyperparameter ranges), with the average ROC-AUC score as the optimization criterion. The hyperparameter tuning was performed exclusively on the training set to limit information leakage. This process was repeated for every feature combination, and the optimal model–feature pairing was selected based on the highest global ROC-AUC score.

#### F. Spatial Refinement

Spatial refinement was applied to improve the classification accuracy by refining the point-level classifier prediction score $p_i$ over the interrogated region. For this, we leveraged the high number of point measurements obtained from an area of interest by spatial averaging each FLIm point measurement with both inverse distance and SNR weighting. The spatial averaging of point measurements allowed the rectification of isolated point-level misclassifications. To preserve the lateral resolution of the prediction map, the averaging was limited to a 15-pixel radius (approximately 0.55 mm) of at least five point-measurement centers.

#### G. Global and Local Model Interpretation

A two-tier interpretability framework with Shapley Additive Explanations (SHAP)[24] is used to enhance clinical interpretability and inform improvements in data acquisition and processing. This framework integrates both global and local model explanations. The global interpretation step identifies which FLIm-derived features most consistently drive decision-making across the entire dataset, while the local interpretation step examines how individual features influence prediction outcomes (i.e., classifying cancerous vs. healthy tissue) at the point-measurement level.

For the global interpretability analysis, we generated SHAP summary plots to aggregate the absolute Shapley values across all FLIm points, highlighting the most influential predictive features and patterns. Conducting local interpretability for every single point is cumbersome, given that each FLIm scan can contain on the order of 5,400 points or more. To address this complexity, we performed local interpretation on a subset of indexed points selected based on the label quality score derived from a confidence learning approach.

Specifically, each FLIm point was assigned to one of four categories (indicating potential low confidence relative to ground truth) using a quartile-based subsampling strategy informed by a label quality score. From each category, we then selected up to 12 representative points (where sufficient points were available) for local SHAP analysis. This targeted subsampling offers a practical method to explore how features contribute at the point level, while retaining a manageable scope for in-depth evaluation.

#### H. Experiment setup and Evaluation

To evaluate and benchmark the classification accuracy, the performance of the data-centric model was compared against other training strategies, including agnostic (i.e., trained with all the data) and anatomic (i.e., trained with anatomy-specific information) approaches. All three approaches were optimized for model and feature selection as detailed in Table IV. The classification model was evaluated at both the point-measurement and tissue-region levels, with the latter enabling assessment of performance across an entire tissue surface. A leave-one-patient-out cross-validation (LOPO-CV) approach was employed. Model validation was performed using the area under the receiver operating characteristic curve (ROC-AUC). Both global ROC-AUC (across the entire dataset) and

Table. IV Hyperparameter specifications derived from Bayesian-optimized grid search. The XGBoost was selected as the highest-performing classifier for all three model strategies for the FLIm feature set: Average Lifetime, Intensity Ratio, and Laguerre Coefficients from spectral channels 1–3. Refer to supplementary: Fig. S1 contains the summary results of grid search.

| Model | No. Estimators | Max Depth | Learning Rate |
|---|---|---|---|
| Agnostic | 135 | 3 | 0.037 |
| Anatomic-Base of Tongue | 114 | 7 | 0.029 |
| Anatomic-Palatine Tonsil | 81 | 3 | 0.032 |
| Anatomic-Oral Tongue | 80 | 3 | 0.091 |
| Data Centric | 78 | 3 | 0.112 |

patient-level ROC-AUC (for patients with both classes present and at least 100 samples per class) were calculated. To account for the dataset imbalance across anatomical regions, an optimal threshold, derived by maximizing Youden's index from the anatomy-based ROC-AUC, was applied as the decision boundary to compute the sensitivity and specificity.

### V. Acknowledgements

The authors thank Dr. Brent W. Weyers and Dr. Katjana Ehrlich for their contributions to the creation of the dataset. The authors also thank Randev Sandhu, CCRP, Clinical Research Coordinator at the University of California, Davis Medical Center, Department of Otolaryngology, for assistance with patient enrollment and consent, maintenance of patient records and medical histories, and dissemination of study information to the research team.

## Supplementary Files

This is a list of supplementary files associated with this preprint. Click to download.

- ManuscriptDCHNCv3Supplementary.docx
